# Cranial Pathologies in a Specimen of *Pachycephalosaurus*

**DOI:** 10.1371/journal.pone.0036227

**Published:** 2012-04-30

**Authors:** Joseph E. Peterson, Christopher P. Vittore

**Affiliations:** 1 Department of Geology, University of Wisconsin Oshkosh, Oshkosh, Wisconsin, United States of America; 2 Department of Radiology, Rockford Memorial Hospital, Rockford, Illinois, United States of America; Raymond M. Alf Museum of Paleontology, United States of America

## Abstract

**Background:**

A frontoparietal dome of a large pachycephalosaurid collected from the Upper Cretaceous Hell Creek Formation in 2001 is identified as *Pachycephalosaurus wyomingensis*. The specimen features two large oval depressions on the dorsal surface, accompanied by numerous circular pits on the margin and inner surface of the larger depressions.

**Methodology/Principal Findings:**

In order to identify the origin of these structures, computed tomography (CT) data and morphological characteristics of the specimen are analyzed and compared with similar osteological structures in fossil and extant archosaurs caused by taphonomic processes, non-pathologic bone resorption, and traumatic infection/inflammatory origins. The results of these analyses suggest that the structures are pathologic lesions likely resulting from a traumatic injury and followed by secondary infection at the site.

**Conclusions/Significance:**

The presence of lesions on a frontoparietal dome, and the exclusivity of their distribution along the dorsal dome surface, offers further insight into frontoparietal dome function and supports previously hypothesized agonistic behavior in pachycephalosaurids.

## Introduction

Pachycephalosaurs (Ornithischia: Marginocephalia) [1], [2] are a group of bipedal ornithischian dinosaurs characterized by their distinctive, greatly thickened cranial dome composed of the fusion and expansion of the frontals and parietals [3]. These domes are typically surrounded with ornamental spikes that vary in size among taxa.

Frontoparietal domes have been hypothesized to have served as courtship weapons for agonistic competition, much like the observed behavior in modern Caprinae [4]. This hypothesis has been supported by finite-element modeling of the physical capabilities of different taxa of pachycephalosaurids, illustrating their ability to withstand considerable impact forces [5], [6].

The identification of expected injuries, such as fractures and depressions sustained during such high-impact bouts are conspicuously absent in the pachycephalosaurid literature [5], [7], [8]. While pathologic features have been speculated in some pachycephalosaurid specimens, such as the holotypes of *Gravitholus albertus* and *Texacephale langstoni* [9], [10], and unidentified frontoparietal domes from the San Carlos and Aguja Formations [11], such claims have been alternatively speculated as taphonomic artifacts [12] or as a result of non-traumatic bone resorption [13].

This study describes a large frontoparietal dome of *Pachycephalosaurus wyomingensis* collected from the Upper Cretaceous Hell Creek Formation (Latest Maastrichtian) of Carter County, Montana in 2001. During preparation, the specimen was found to exhibit two large depressions on the dorsal surface, accompanied by numerous circular pits on the margin and inner surface of the larger depressions. Based on computed tomography (CT) data analyses and comparisons with similar structures in extant and fossil archosaurs, these features are interpreted as bone pathology likely resulting from trauma. Because the features present on this frontoparietal dome specimen are well preserved, and pathology has not been previously described in *Pachycephalosaurus*, this specimen merits a brief description.

## Materials and Methods

### Locality

The specimen was collected in Carter County, Montana, USA, approximately 60–70 meters above the base of the Hell Creek Formation (Latest Maastrichtian), which averages 150 meters in thickness in southeastern Montana [14]. Exact coordinates of the collection site are on file at the Burpee Museum of Natural History (BMR). The frontoparietal dome was found at the base of a mudstone butte; no other skeletal remains were recovered.

### Description

**Figure 1:**
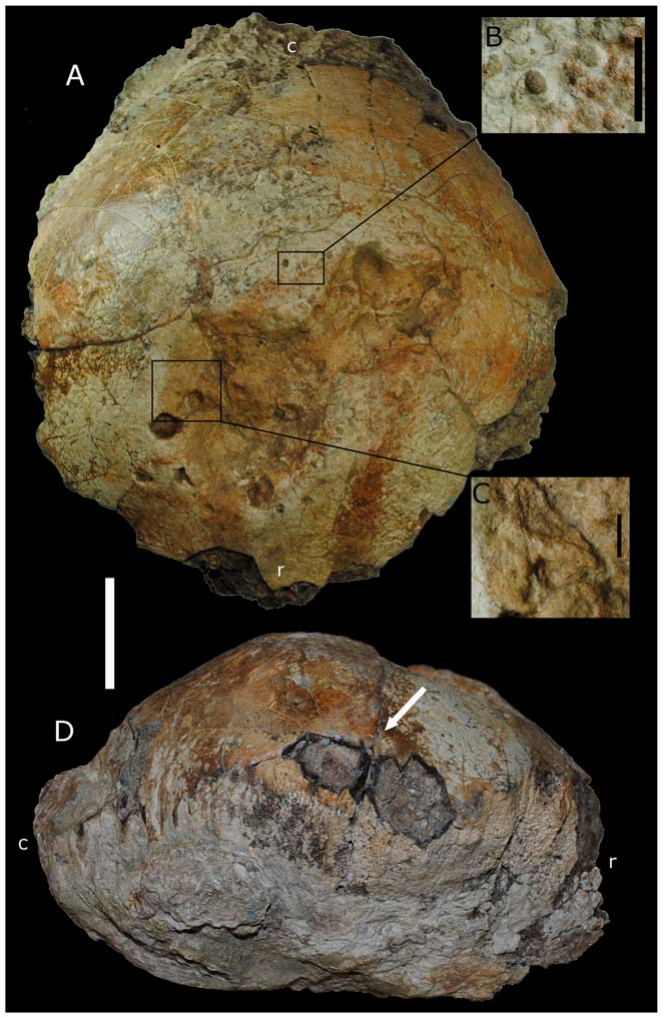
BMR P2001.4.5. (A) Dorsal view of calvaria. Scale bar equals 5 cm. (B) Close-up of the peripheral lesions external to the larger depressions. Scale bar equal 10 mm. (C), Close-up of the marginal slope of the rostral-most depression. Scale bar equals 10 mm. (D) Right lateral side of BMR P2001.4.5 showing exposure of the frontoparietal suture (arrow). r = rostral, c = caudal.

The specimen, BMR P2001.4.5, consists of an isolated frontoparietal dome (Figure 1). The specimen measures 620 mm in circumference, 310 mm in length from the rostral end to the posterior margin of the caudal region, and 132 mm in height. This is consistent with the average dome dimesions of the Maastrichtian pachycephalosaurid *Pachycephalosaurus wyomingensis*, in which adult frontoparietal domes average between 260 and 360 mm in length [15]. The frontoparietal suture is present on the right lateral side beneath the cortical surface (Figure 1D), though no suture is apparent on the dorsal surface of the specimen.

Four nominal taxa of pachycephalosaurids have been described from the Hell Creek Formation: *Pachycephalosaurus wyomingensis* (Brown and Schlaikjer, 1943) [16], *Stygimoloch spinifer* (Galton and Sues, 1983) [17], *Dracorex hogwartsia* (Bakker et al., 2006) [18], and *Sphaerotholus buchholtzae* (Williamson and Carr, 2002) [19]. The dorsal surface of BMR P2001.4.5 is smooth, which is a characteristic of the largest pachycephalosaurid specimens [16] and lacks the distinctive, regular dimpling found in *Sphaerothlous* or polygonal sulci present on smaller domes [19]. Considering its large size, BMR P2001.4.5 is too broad and large to be attributed to *Stygimoloch*, which possesses a smaller, mediolaterally narrow dome [8]. However, due to the large frontoparietal dome of *Pachycephalosaurus wyomingensis* among other pachycephalosaurids, the large size of BMR P2001.4.5 suggests the specimen is referable to *Pachycephalosaurus* [16].

Previous studies have called the validity of *Stygimoloch* and *Dracorex* into question, suggesting that they are younger ontogenetic stages of *P. wyomingensis* [15]. Furthermore, Goodwin and Horner [15] have suggested that variation in frontoparietal surface texture may be a factor of ontogeny and dome development. Regardless of this debate, the nearly complete fusion and closure of the frontoparietal suture, the massive size of BMR P2001.4.5, and lack of dorsal surface texturing or sulci suggest that it is a mature individual and thus maintains its identification as *Pachycephalosaurus wyomingensis*.

### Systematic Paleontology

Dinosauria Owen, 1842 [20]

Ornithischia Seeley, 1887 [21]

Pachycephalosauria Maryanska and Osmolska, 1974 [22]

Pachycephalosauridae Sternberg, 1945 [23]

Pachycephalosaurinae Sereno, 2000 [2]

*Pachycephalosaurus wyomingensis* Brown and Schlaikjer, 1943 [16]

### Bone Pathology

**Figure 2 pone-0036227-g002:**
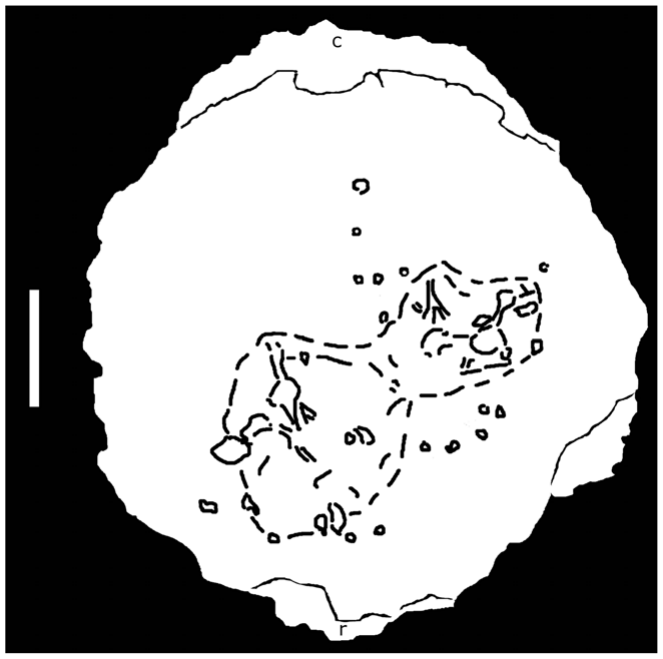
An index drawing of the distribution of lesions. Scale bar equal 5 cm. r = rostral, c = caudal.

**Figure 3:**
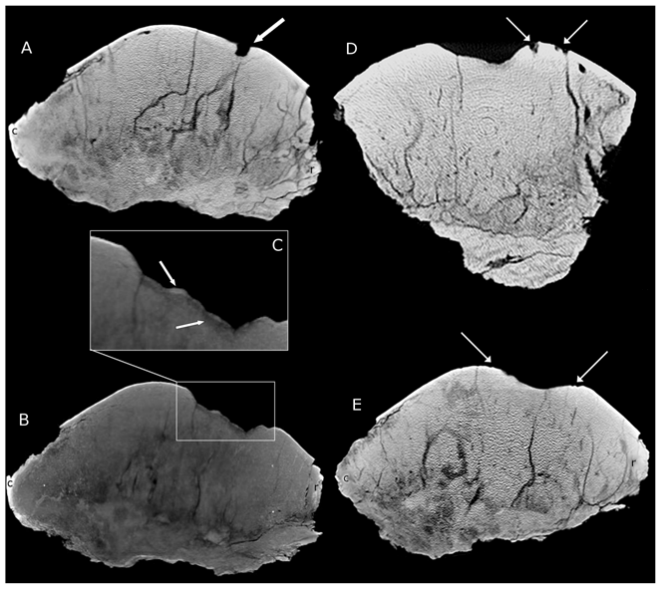
CT images of BMR P2001.4.5. A. Sagittal image showing a focal, deep penetrating lesion in the dorsal surface. B. Sagittal MIP (maximum intensity projection) reformatted image of the irregular surface of the large depressions. C, The surface of the depression possesses a high-density surface above a lower density band. D, Coronal, and E, sagittal images of the peripheral lesions at the margin of the larger central pathologies on the dorsal surface. r = rostral, c = caudal.

A series of small erosive pits and two large crateriform depressions occur on the dorsal surface of BMR P2001.4.5 (Figures 1, 2). The two large depressions are positioned on the frontal region of the dorsal surface, measure approximately 5 cm in diameter, and have a maximum depth of 1.6 cm. The inner surface and marginal rim of the depressions have several smaller circular pits (Figures 1B, 3A, D) ranging from 1 to 10 mm in diameter and depth, the more shallow of which possess smooth surfaces. The larger pits flare where they penetrate into the dome, resulting in a flask-shaped cross section (Figure 3A), and possess horizontally oriented canals that extend into the larger depressions (Figures 1A, 3D). The larger and deeper structures present on BMR P2001.4.5 penetrate the outer-most cortex of the dorsal surface, exposing an irregular area of compact bone immediately above a lower-density band (Figure 3B, C).

Pachycephalosaurid frontoparietal domes possess a variety of histological zones dependent on genus and ontogenetic stage [24]. The middle and least dense of these zones, Zone II, occurs in subadult individuals, and increases in density throughout ontogeny. Coring or thin sectioning of BMRP 2001.4.5 was not permitted; however a series of computed tomography (CT) scans were conducted to gain further insight into the ontogenetic stage and depression characteristics within the specimen. The scan was performed with an Aquilion Toshiba 64-Slice CT scanner at Rockford Memorial Hospital in Rockford, IL, and conducted at settings for medical diagnoses of bone pathology (135 kV, 300 mA, 0.5 mm pixel resolution, and 0.5 mm thickness). The significant thickness and very high density of BMR P2001.4.5 caused considerable noise in the CT images, which decreased some of the examination detail. However, this did not prevent examination and characterization of the important structural features of the depressions. The raw CT data are archived at the Burpee Museum of Natural History. The frontoparietal dome was also digitized into a 3D model using a NextEngine Desktop 3D Scanner (Figure S1).

While histologic sections could not be produced for this study, BMR P2001.4.5 was compared with similar osteological structures in fossil and extant archosaurs in order to infer the cause of the depressions and erosive pits. Explored causes include taphonomic alteration (i.e. insect modification and bone weathering), non-traumatic bone resorption, and traumatic origins (i.e. injury and subsequent infection).

## Results

### Taphonomic Alteration

Taphonomic alteration resulting from biostratinomic and diagenetic processes is commonly responsible for erosional features in bone [25]–[27]. Indentations and borings in bones due to early biostratinomic processes such as scavenging [28] and insect modification [26] are common in the vertebrate fossil record. However, such features can usually be attributed to these origins by specific traces such as parallel puncture and drag marks in scavenged bones [29] and mandible marks from insect modification [26]. The absence of these traces on BMR P2001.4.5 suggests that post-mortem scavenging and insect damage are not likely etiologies.

Previous occurrences of erosive and pitted structures on pachycephalosaurid crania have been regarded as a result of taphonomic weathering and erosion due to subaerial exposure. Tanke and Farke [13] noted possible pitting in frontoparietal domes of *Stegoceras*, but suggested the features might have been due to water-wear. Sullivan [30] also commented on the possibility of taphonomic effects as cause for features present in the holotypes of *Gravitholus albertus* and *Prenocephale edmontonensis*. The effects of weathering and erosion on both ancient and modern bone have been thoroughly described [25], [31]–[33]. Parallel cracks and polygonal fracture patterns commonly characterize subaerially exposed and eroded bone surfaces [25], [33]. The surface of BMR P2001.4.5 lacks these characteristics, suggesting that the depressions and pits are not likely the result of extensive subaerial exposure and erosion.

### Non-Traumatic Bone Resorption

Lesions appearing in the squamosal fenestrae of numerous specimens of chasmosaurine ceratopsids have been attributed to bone resorption [13]. Bone remodeling or “punched out lesions” (POLs) described in ceratopsians usually occur in thin regions of the squamosal, on both internal and external bone surfaces, and the lesions exhibit smooth surfaces [13]. The structures on BMR P2001.4.5 exhibit irregular lesion surfaces that extend into deeper bone tissues, unlike the smooth surfaces in POLs. Furthermore, the massive size of BMR P2001.4.5 is also inconsistent with POLs, such as those commonly seen in thin ceratopsian squamosals and parietals. This suggests that non-traumatic bone resorption is not a likely source of the depressions.

### Trauma

Lesions in bone due to injury and disease have been well documented, and may best explain the origins of the lesions in BMR P2001.4.5. Osteomyelitis is the infection of bone and bone marrow and can be classified as acute, subacute or chronic. Chronic osteomyelitis is a severe, persisting infection of bone and bone marrow, and can be the result of trauma to the bone itself or spread from adjacent soft tissue infection. An anatomic classification of chronic osteomyelitis has been proposed by Cierny and Mader [34] (Table 1).

**Table 1:** Anatomic classification of chronic osteomyelitis (After Cienry and Mader, 1984).

**Type 1**	Endosteal or medullary lesion.
**Type 2**	Superficial osteomyelitis limited to the surface.
**Type 3**	Localized, well-marked lesion with sequestration and cavity formation.
**Type 4**	Diffuse osteomyelitis lesions.

The acute phase of osteomyelitis generally does not show radiographic abnormalities. Findings of acute or subacute osteomyelitis, which is commonly bacterial or fungal in origin, include periosteal reaction, cortical irregularity and demineralization, while findings of chronic osteomyelitis include thick, sclerotic, irregular bone, and an elevated periosteum [35].

If a wound is present and shows signs of infection, then any exposed bone in the bed of the wound is considered infected. An open fracture, one in which there is bone exposed to the outside environment, is at increased risk for development of superimposed infection. The amount of overlying soft tissue on pachycephalosaurid domes is unknown but the supposition that it was minimal [5] would lead to a higher likelihood of direct contamination. Subsequent ongoing exposure to the surrounding environment would logically provide additional opportunity for contamination.

Open fractures in the human medical literature are classified based on the mechanism of injury, degree of soft tissue damage, fracture configuration and level of contamination [36]; the incidence of infection is influenced by the type of open fracture. The lesions present on BMR P2001.4.5 correlate well with the higher level of the classification scheme, Type III – B in which the fracture is associated with prominent injury to (or loss of) soft tissue, periosteal stripping and exposure of bone, and massive contamination [37]. Any possible comminuted fracture fragments could have left the fracture site immediately as the result of the force applied or could have fallen from the parent bone in a delayed fashion. Over time, infected bone fragments (i.e. sequestra) can be ejected from a site of chronic osteomyelitis through a cloaca, or resorbed [35].

The CT images show chronic changes involving the surface of the concave bone lesion where the margins are smooth and rounded compatible with healing (Figure 3B–E). The expected thick, sclerotic nature of bone tissue seen with chronic osteomyelitis may be masked by the nature of the particular bone anatomy for pachycephalosaurids [22]. However, the expected irregularity of the floor of the lesion is well demonstrated in BMR P2001.4.5. These irregular floors show a varying thickness of higher density bone over a rarefied zone which does not exist at the same depth elsewhere in the specimen, consistent with woven osseous remodeling and overlying healing new bone (Figure 3C). A few small rounded concavities penetrate the bone surface at the periphery of the larger defect (Figures 3D, E). Their more focal nature is characterized by acute margins consistent with localized erosions. These characteristics suggest the lesions are of traumatic etiology with superimposed, ongoing infection.

## Discussion

Evidence of trauma and pathology are relatively common on dinosaur bones and are frequently used for behavioral inferences [29], [38], [39]. Head-butting behavior modeled after comparable interactions observed in caprids and other bovids has popularly been proposed for pachycephalosaurids [40] and has been supported by structural models [5], [6]. However, pathological evidence for such behavior has not been previously described in pachycephalosaurids [5].

Extant birds and crocodilians commonly exhibit osteological lesions following. In such cases, trauma or contraction of disease can cause the accumulation of fibrous, caseous necrotic masses due to infection, and the isolated infection results in the removal and modification of bone [41]. While these kinds of infections can arise from a variety of causes, the large depressions present on BMR P2001.4.5 appear similar to injuries sustained from compression fractures and blunt force trauma in extant birds and crocodilians [41]–[43].

While no extant archosaurs have structurally comparable frontoparietal domes like pachycephalosaurids, intraspecific head-butting behaviors have been documented in some extant archosaurs, such as the Helmeted Hornbill (*Buceros vigil*) and the Southern Cassowary (*Casuaris casuaris johnsonii*) [44], [45]. Similar head-slapping behaviors among crocodilians during intra- and interspecific conflicts have also been extensively documented [46]. In many cases, conflicts occur among juveniles who engage in intra- and interspecific disputes that commonly result in injury and occasionally infection [46]–[49]. Kicking and facial pecking during intraspecific bouts among ostriches has been shown to result in a high frequency of injuries [42].

**Figure 4:**
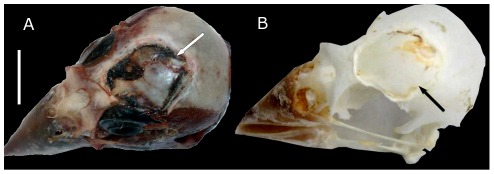
Cranium of European Greenfinch (*Carduelis chloris*) after surviving impact with a window. A. Greenfinch head without skin, B. Greenfinch skull with a healed depressed fracture. Scale bar equals 1 cm. Images courtesy of Johs Erritzøe (www.birdresearch.dk).

Common injuries in extant birds also include impact fractures to the skull due to collision with man-made structures such as windows and buildings. While these injuries are often fatal, instances of survival have been documented [42], [43]. For example, specimens of Rocky Mountain Grosbeak (*Hedymeles melanocephalus papago*) and European Greenfinch (*Carduelis chloris*) have exhibited extensive osteological remodeling following a cranial injury [42] (Figure 4). In such instances, the resulting lesions formed large circular depressions with irregular surfaces on the calverium similar to the depressions observed on BMRP 2001.4.5.

The lesions in BMR P2001.4.5 are clustered over the thickest region of the dome (Figures 1–3); this distribution corresponds well to the location of expected traumatic pathologies resulting from agonistic behavior proposed for pachycephalosaurids [5], [7]. While a formal study on pathologies in pachycephalosaurids has yet to be conducted, it is possible that pathologic lesions such as those in BMR P2001.4.5 may have been previously misidentified as taphonomic artifacts and non-traumatic bone resorption in other specimens. A re-evaluation of previously described frontoparietal domes is likely to yield more pathologic dome specimens; the holotypes of *Gravitholus albertus* and *Texacephale langstoni* also possess similar cranial features exclusively on the dorsal surface (author's pers. observ.). The occurrence of cranial lesions in pachycephalosaurids has considerable implications for behavior and frontoparietal dome function beyond species recognition [50], and provide further evidence of agonistic behavior in pachycephalosaurs.

## Supporting Information

Figure S1 **3D model of BMRP 2001.4.5.** Model was created with the NextEngine 3D Desktop scanner and software, converted to U3D using Meshlab, and assembled in a *.pdf with Basic MikTex.
(PDF)
