# Supplementary material for: Cranial Pathologies in a Specimen of Pachycephalosaurus
Source: PLoS One. 2012 Apr 30;7(4):e36227. doi: 10.1371/journal.pone.0036227 (PMC3340332; doi:10.1371/journal.pone.0036227)
Supplement: Figure S1 — 3D model of BMRP 2001.4.5. Model was created with the NextEngine 3D Desktop scanner and software, converted to U3D using Meshlab, and assembled in a *.pdf with Basic MikTex. (PDF) [file pone.0036227.s001.pdf]

BMR P2001.4.5 Pachycephalosaur Frontoparietal Dome

See manuscript text for measurements and dimensions

12th January 2012

temp.u3d

3D model made with the NextEngine 3D Desktop scanner and sofware, converted to U3D using Meshlab and finally assembled in a pdf with Basic Miktex.
